# Factors associated with seeking post-abortion care among women in Guangzhou, China

**DOI:** 10.1186/s12905-020-00980-0

**Published:** 2020-06-10

**Authors:** Hui Wang, Yan Liu, Ribo Xiong

**Affiliations:** 1 Department of gynecology &obstetrics, The Third Affiliated Hospital of Southern Medical University, Guangzhou, China; 2 Department of rehabilitation, Nanhai Hospital, Southern Medical University, Foshan, China

**Keywords:** Post-abortion care, Contraception, Utilization, Women, China

## Abstract

**Background:**

In China, the vast majority of induced abortions are performed in public hospitals. However, post-abortion care (PAC) services are provided through the national network of family planning clinics, which are independent of the health care system. The integration of PAC services into abortion clinics in public hospitals is a new concept. This study aimed to assess PAC utilization among abortion patients, and identify the possible factors associated with PAC uptake in Guangzhou, China.

**Methods:**

A cross-sectional survey was conducted among 431 women aged 15–43 years in Tianhe district of Guangzhou, China from June to September 2018. We estimated multivariate logistic regression model to examine the factors associated with utilization of PAC services.

**Results:**

Less than half (42%) of the participants used PAC services. Married women were 2.7 times significantly more likely to use PAC services than their unmarried counterparts. Immigrants were 52% significantly less likely to use PAC services than non-immigrants. Women who perceived that their fertility could return later and those who did not know were 45 and 61% significantly less likely to use PAC services compared to those who knew that their fertility could return soon after an abortion. Women with limited decision-making autonomy regarding contraceptive use were 54% significantly less likely to use PAC services than those who made such decisions themselves.

**Conclusions:**

The findings suggest the need for policies and programs to not only strengthen the provision of PAC services but also promote uptake among disadvantaged sub-groups of women in the study setting.

## Background

Unplanned pregnancies and induced abortions (IAs) remain an important public health issue, especially among women from developing countries. IAs result from unplanned pregnancies which occur due to contraceptive failure or non-use of contraception during sexual intercourse. Estimates suggest that the average IA rate is approximately 58 per 1000 women worldwide [1]. In China, IA is a legal procedure in the first trimester of pregnancy. Recent estimates show that between 6 and 9 million IAs occur in the country every year [2]. Nearly half of low-income urban women in the country had experienced IAs with 31% being repeated abortions [2]. The causes of IAs are complicated. One of the major reasons is lack of quality post-abortion care (PAC) counseling [2].

The World Health Organization recommends counseling on and provision of contraceptive methods to all those women who wish to prevent unintended pregnancies and subsequent IA [3]. The term PAC was first used in 1991 and referred to an approach to break the cycle of unwanted pregnancy and improve women’s sexual as well as reproductive health [4]. The integration of family planning (FP) counseling and method provision into abortion services is an essential part of PAC. Multiple studies have demonstrated the effectiveness of PAC in reducing repeat IAs when offered to abortion patients prior to discharge from health facilities [5, 6]. However, knowledge of the extent of PAC utilization is limited. Available data regarding PAC utilization rate is varied across countries and regions. How the specific context affects the implementation of PAC and what factors contribute to the accessibility of PAC services continuously attract researcher’s interest.

Recent studies suggest that type of health facility, the decision maker on timing to have a child, knowledge of fertility return after abortion and husband’s attitudes towards contraceptives may be significantly associated with PAC utilization [7–9]. The existing literature mainly focused on abortion patients in African countries which have significant disparities in culture and social environment from China. Report on the status of PAC utilization among Chinese abortion patients is scant and inconclusive. Available research uses qualitative methods including semi-structured interviews and focus group discussion were employed to explore the feasibility and acceptability of high quality PAC services from the service providers’ perception [10]. This is a limitation given the rapidly changing demographics of abortion patients in the south area of China over the past decade and inability to generalize findings from such studies to the region.

In China, the vast majority of IAs are performed in public hospitals. However, PAC services are provided through the national network of family planning clinics, which are independent of the health care system. The integration of PAC services into abortion clinics in public hospitals is a new concept. In 2010, the National Population and Family Planning Commission launched a pilot program to include the provision of PAC into 486 hospitals [11]. Guangzhou, where this study was conducted, was one of the pilot cities where free PAC services were provided in selected public hospitals. There is, however, limited understanding of the extent to which PAC services are utilized among abortion patients in such settings. Furthermore, factors that may be associated with PAC utilization are not well-understood. Hence, this study aimed to assess post-abortion care (PAC) utilization among abortion patients, and identify the possible factors affecting PAC uptake in Guangzhou, China.

## Methods

### Study setting

Guangzhou is the capital city of Guangdong province, an economically developed province in south China. Tianhe district, where this study was conducted, was the pilot site where free PAC services were offered in public hospitals.

### Study design

A cross-sectional survey was conducted in Tianhe district of Guangzhou during the period of June to September 2018. All those women who had experienced at least once IA in the selected public hospitals were included in the study. However, we excluded women who were unable to speak or listen and those with psychiatric disorder.

### Sample size determination

The sample size was determined using the single population proportion assuming 57.4% of women utilizing PAC services and the desire to obtain reasonable estimates at 95% confidence level and 5% margin of error [12].

The total sample size was 431 women, taking into account 15% non-response.

### Sampling procedure

Three public hospitals in Tianhe district that provided PAC services on a pilot basis were included in the study. The average number of PAC users was estimated according to the recent quarterly report of patients flow in each hospital. We used a systematic random sampling approach to select participants. The number of participants from each hospital was determined based on population proportion to size. Thus the number of participants was 156, 127 and 148 from the first, second and third hospital respectively.

### Data collection

Data were collected using an anonymous structured questionnaire. The development of the questionnaire was informed by existing literature on PAC utilization. The questionnaire was reviewed by experts in reproductive medicine and clinical epidemiology from China. After the pilot test, the questionnaire was revised accordingly, covering the following information: 1) socio-demographic characteristics, such as age, marital status, education, employment status, income and migrant characteristics; 2) reproductive history such as parity, previous IAs, number of living children; 3) contraceptive and reproductive health knowledge such as knowing how soon fertility returns and the time of getting pregnant again, uptake and method of contraception; 4) other related variables such as husband’s/partner’s attitude towards contraceptive use and the person responsible for making contraceptive decisions.

All participants were interviewed face-to-face after obtaining written permission. They were assured of the confidentiality of their identity and responses. The data collection phase was completed with the help of seven post-graduate female nurses. They were trained for 2 days by the principal investigator covering interview techniques, quality control, completeness of information and research ethics. All completed questionnaires were checked for completeness and consistency.

### Statistical analysis

The primary data was entered into Epidata 3.1 before being exported to SPSS 20.0.

Women with missing information on key attributes were excluded from the analysis.

Categorical variables were presented as counts and proportions. Cross-tabulations with Chi-square test were used to assess significance of differences in socio-demographic characteristics, reproductive history, reproductive health knowledge and other related factors between abortion patients who used PAC services and those who did not. The independent variables that were significantly associated (*p*<0.05) with PAC services utilization in cross-tabulations were considered as possible contributing factors and entered into a multivariate logistic regression model. The model examined factors that were significantly associated with use of PAC services after controlling for possible confounders. The outcome variable in the regression analysis, use of PAC services, was dichotomous (whether the participants had used the services or not). The model controlled for marital status at the time of interview (married and unmarried), place of household registration (whether the participant’s household was registered in Guangzhou or not), knowledge of return to fertility (within 10–14 days, after 3–4 weeks, and don’t know), and the person responsible for making contraceptive decisions (respondent herself, respondent’s husband/partner, or both). Odds Ratios (ORs) with 95% confidence intervals (95% CIs) were calculated to measure the strength of association. Estimates with *p*-values less than 0.05 were considered statistically significant.

### Ethics

The study protocol was approved by the Research Ethics Board of Southern Medical University, China. All participants provided written informed consent before being interviewed.

## Results

### Socio-demographic characteristics of study participants

Of the 431 eligible women, 425 (98.6%) consented to participate in the survey. Among those who consented to participate in the survey, 413 (97%) completed the interviews. Of the 413 abortion patients who completed the interviews, 174 (42%) utilized PAC services.

Slightly more than a quarter (28%) of the participants were aged between 25 and 29 years and a similar proportion (27%) were between the ages of 20 and 24 years. More than half (55%) of the participants were unmarried. More than three-quarters (79%) of the participants had senior high school or college level education. Regarding employment status, 231 (56%) were employed and 125 (30%) were students. The highest proportion (42%) of participants earned between 2000 and 4999 Chinese Yuan per month, followed by those who earned between 5000 and 7999 Chinese Yuan (25%). Distribution by place of household registration shows that 62% of the participants were immigrants (Table 1).

**Table 1:** Socio-demographic characteristics of study participants

Variables	Frequency	Percentage (%)
Age		
15 ~ 19	76	18
20 ~ 24	110	27
25 ~ 29	116	28
30 ~ 34	60	15
≥ 35	51	12
Marital status		
Married	185	45
Not married	228	55
Level of education		
Junior high school or less	87	21
Senior high school	127	31
College or more	199	48
Employment status		
Employed	231	56
Student	125	30
Unemployed	57	14
<2000 CNY	96	23
2000 ~ CNY	174	42
5000 ~ CNY	104	25
≥ 8000 CNY	39	9
Household registration place in Guangzhou		
Yes	157	38
No	256	62

*CNY* Chinese Yuan

### Variations in the use of PAC services by background characteristics

The proportion of married women who used PAC services was significantly higher than that of unmarried women (52 and 31%, respectively; *p* < 0.01; Table 2). The proportion of immigrants that used the services was almost four times lower than that of non-immigrants (19 and 80%, respectively; p < 0.01; Table 2). In addition, the proportion that used the services was significantly higher among women who knew that their fertility would return soon following an abortion (77%) than among those who knew that their fertility would return later (50%) or those who did not know when their fertility would return (10%; *p* < 0.01; Table 4). The proportion was also significantly greater among women who made contraceptive decisions themselves (56%) than among those who made the decisions jointly with their partners (39%) or those whose partners solely made such decisions (30%; p < 0.01; Table 4). There were, however, no statistically significant variations in the proportions using the services by the other factors considered (Tables 2, 3 and 4).

**Table 2:** Variations in the use of PAC services by socio-demographic characteristics

Characteristics	Total	Use of PAC services		χ^**2**^	***P***
		Frequency	Percentage(%)		
Age				0.975	0.914
15 ~ 19	76	31	40.8		
20 ~ 24	110	48	43.6		
25 ~ 29	116	46	39.7		
30 ~ 34	60	28	46.7		
≥ 35	51	21	41.2		
Marital status				156.2	0.000
Married	185	97	52.4		
Not married	228	77	31.3		
Level of education				0.107	0.948
Junior high school or less	87	36	41.4		
Senior high school	127	55	43.3		
College or more	199	83	41.7		
Employment status				0.383	0.826
Employed	231	95	41.1		
Student	125	53	42.4		
Unemployed	57	26	45.6		
Annual monthly income				0.070	0.995
<2000RMB	96	41	42.7		
2000 ~ RMB	174	74	42.5		
5000 ~ RMB	104	43	41.3		
≥ 8000RMB	39	16	41.0		
Household registration place in Guangzhou				151.0	0.000
Yes	157	126	80.3		
No	256	48	18.8

**Table 3:** Variations in the use of PAC services by reproductive history

Reproductive history	Total	Use of PAC services		χ^**2**^	***P***
		Frequency	Percentage(%)		
Parity				0.117	0.943
0	157	67	42.7		
1	136	58	42.6		
≥ 2	120	49	40.8		
Previous induced abortion				0.028	0.921
Yes	195	83	42.6		
No	218	91	41.7		
Number of children alive				0.357	0.836
0	155	68	43.9		
1	136	55	40.4		
≥ 2	122	51	41.8		
Condition of the pregnancy				0.410	0.815
Planned/Wanted	72	32	44.4		
Unplanned/Wanted	125	50	40.0		
Unplanned/Unwanted	216	92	42.6

**Table 4:** Variations in the use of PAC services by reproductive knowledge and other related factors

Reproductive knowledge and other related factors	Total	PAC services utilization		χ^**2**^	***P***
		Frequency	Percentage(%)		
Knowledge on how soon fertility returns and could get pregnant again				128.5	0.000
Within 10 ~ 14 days	116	89	76.7		
After 3 ~ 4 weeks	137	69	50.4		
Don’t know	160	16	10.0		
Any uptake of contraception				0.105	0.949
Yes	188	78	41.5		
No	198	85	42.9		
Missing	27	11	40.7		
Contraceptive use (multiple responses allowed)					
None	82	34	41.5	0.019	1.000
Traditional methods	66	26	39.4	0.241	0.684
IUD	136	56	41.2	0.076	0.832
Oral contraceptives	254	108	42.5	0.041	0.918
Injectables	59	27	45.8	0.372	0.571
Male condoms	299	129	43.1	0.456	0.577
Husbands’/partners’ attitude on contraceptive use				0.042	0.979
Approve	178	76	42.7		
Disapproved	158	66	41.8		
Uncertain	77	32	41.6		
Person responsible for making contraceptive decisions				26.481	0.000
Husband/partner	194	58	29.9		
Women herself	178	100	56.2		
Both	41	16	39.0		

Traditional methods: rhythm, lactational amenorrhea, and withdrawal; *IUD* intrauterine device

### Factors associated with PAC utilization

Table 5 shows the factors associated with PAC utilization among abortion patients in the study site. Married women were 2.7 times significantly more likely to use PAC services compared to those who were unmarried (OR = 2.713, 95% CI: 1.734 ~ 2.996). In relation to place of household registration, immigrant women were 52% significantly less likely to use PAC services compared to native women (OR = 0.483, 95% CI: 0.203 ~ 0.716). Similarly, participants who knew that their fertility could return 3 ~ 4 weeks post-abortion were about 45% significantly less likely to utilize PAC services compared to women who knew that their fertility could return within 10 ~ 14 days (OR = 0.545, 95%CI: 0.308 ~ 0.802). Those who were uncertain about when their fertility could return were also 61% significantly less likely to receive PAC services compared to those who knew that their fertility could return within 10 ~ 14 days (OR = 0.391, 95%CI: 0.294 ~ 0.617). The likelihood of using PAC services also varied by the person responsible for making contraceptive decisions. Women whose husbands were the main decision-makers regarding contraceptive use were 54% significantly less likely to use PAC services compared to those who made such decisions themselves (OR = 0.460, 95% CI: 0.272 ~ 0.809).

**Table 5:** Odds ratios from multivariate logistic regression examining factors associated with PAC utilization in Guangzhou, China

Covariates	***OR***	***SE***	***95%CI***	***P***
Marital status				
Married	2.713	0.108	1.734 ~ 2.996	0.000
Not married	–	–	–	–
Household registration place in Guangzhou				
Yes	–	–	–	–
No	0.483	0.491	0.203 ~ 0.716	0.000
Knowledge on how soon fertility returns and get pregnant again				
Within 10–14 days	–	–	–	–
After 3–4 weeks	0.545	0.086	0.308 ~ 0.802	0.000
Don’t know	0.391	0.235	0.294 ~ 0.617	0.000
Person responsible for making contraceptive decisions				
Woman herself	–	–	–	–
Husband/Partner	0.460	0.098	0.272 ~ 0.809	0.000
Both	0.638	0.137	0.591 ~ 0.910	0.096

## Discussion

The study aimed to determine the proportion of women who utilized PAC services among abortion patients and the factors associated with use of such services in public health facilities piloting the provision of services in Guangzhou, China. The findings show that less than half (42%) of women seeking abortion services in the pilot facilities used PAC services. Uptake of PAC services in the study setting was comparable to the level observed in east region hospitals (43%) but lower than the levels in middle and west region hospitals in the country (72 and 58%, respectively) [13]. This disparity could be due to the variations in the provision of PAC in public hospitals. In China, FP counseling, an integral component of PAC, is provided through the national network of FP clinics, which are almost independent of the health care system. However, the vast majority of IAs are performed in public hospitals. The integration of PAC services within public hospitals is a new concept. The pace with which hospitals in different settings take up the provision of the services may vary depending on available financial, human and infrastructural resources. Policy support in the form of regulations and guidelines, human resources and financial resources are the main considerations regarding the implementation of PAC services. Low uptake of PAC services in the study could also be due to the fact that most (60%) of the participants were internal immigrants. In particular, allocation of healthcare resources is based on household registration, and immigrants do not have the same rights and benefits to services as local registered residents.

The findings of this study show that married women were significantly more likely to use PAC services compared to their unmarried counterparts, which is consistent with the findings of another study conducted in China [8]. However, another study in Ethiopia found that married women were less likely to use PAC services compared to unmarried women [7]. Variations in uptake of PAC services by marital status may depend on the extent to which partners have influence over women’s access to services. Married women may be less likely to seek services in settings where men wield great control over decision-making and resources for seeking care. In contrast, they are likely to seek care in settings where they have control over such resources. Greater use of PAC services among married compared to unmarried women suggests the need for policies and programs targeting unmarried women who often face unmet family planning needs. This could include information, education and communications interventions on sexuality and prevention of unintended pregnancy.

We also found a significant association between household registration place and PAC utilization. Immigrant women were significantly less likely to utilize PAC services compared to their native counterparts. Because medical insurance system in China is based on household registration, immigrants have much less reimbursement in medical expenses such as PAC counseling than the natives in their host cities. In addition, in China, this group tends to be relatively uneducated, in low-income groups, insecure jobs and are excluded from municipal welfare structures in their host cities [14]. The finding suggests that local governments need to promote social integration of immigrants in order to improve access to health services across all segments of the population.

Another key finding of the paper is that women who perceived that their fertility would return later and those who did not know when their fertility would return were significantly less likely to use PAC services compared to those who knew that their fertility would return soon. This is consistent with findings from a study conducted in Ethiopia [7]. Women who knew that their fertility could return soon are likely to take steps to avoid unintended pregnancy, including seeking FP services, compared to those who did not think or know that they could get pregnant soon after having an abortion. The finding suggests the need for improved counseling of clients on the risks of pregnancy following an abortion.

The findings of the study further show that women whose husbands were the main decision makers regarding contraceptive use were significantly less likely to use PAC services compared to those who made such decisions themselves. A study in Nigeria concluded that reluctance to use PAC services was due to husband disapproval [15]. Opposition by the husband could also be due to limited knowledge of women’s reproductive health needs or gendered power differences in control of household resources and decision-making process that favor men. In most hospitals of China, male partners are not allowed to access the department of gynecology and obstetrics. Although they are eventually allowed into FP counseling room in some cases, they are just given some materials regarding reproductive health. Future research could explore whether targeting and including male partners in the provision of PAC could have an impact on increasing PAC utilization among abortion patients.

This study has some limitations. First, the cross-sectional nature of the study did not allow for establishing causal relationships between PAC use and the factors associated with it. Second, the findings may be affected by response bias. Given the sensitivity of the topic, respondents might feel shy to reveal some personal details or may provide responses they feel are socially desirable. Third, the findings might not be representative of abortion patients in China, since participants were recruited from Guangzhou, China.

## Conclusions

Uptake of PAC services still remains low in the study setting where the provision of the services in public hospitals was being piloted. In addition, uptake of the services remains low among disadvantaged sub-groups, including unmarried women, immigrants, those with limited knowledge about bodily functions, and women with limited decision-making autonomy. The findings suggest the need for policies and programs to not only strengthen the provision of the services but also promote uptake among disadvantaged sub-groups of women in the study setting.

## Data Availability

The datasets used and/or analyzed during the current study are available from the corresponding author on reasonable request.

## Abbreviations

IA: Repeat induced abortion
FP: Family planning
PAC: Post-abortion care
